# Biological and Chemical Management of *Aspergillus carbonarius* and Ochratoxin A in Vineyards

**DOI:** 10.3390/toxins16120527

**Published:** 2024-12-06

**Authors:** Maria K. Iliadi, Maria Varveri, Dimitrios I. Tsitsigiannis

**Affiliations:** Department of Crop Science, Laboratory of Plant Pathology, Agricultural University of Athens, Iera Odos 75, 11855 Athens, Greece; maria_iliadi90@hotmail.com (M.K.I.); varvmar@gmail.com (M.V.)

**Keywords:** biopesticides, fungicides, biostimulants, IPM, *Aspergillus carbonarius*, ochratoxin-A, vineyards, grapes

## Abstract

Ochratoxin A (OTA) is a widely distributed mycotoxin and potent carcinogen produced by several fungal genera, but mainly by *Aspergillus carbonarius*. Grape contamination occurs in vineyards during the period between veraison and pre-harvest, and it is the main cause of OTA’s presence in wine. The aim of the current study was the evaluation of 6 chemical and 11 biological plant protection products (PPPs) and biocontrol agents in commercial vineyards of the two important Greek white wine varieties cv. Malagousia and cv. Savatiano. The PPPs were applied in a 4-year vineyard study as single treatments or/and in combinations as part of IPM systems. Subsequently, nine strains of *Aspergillus carbonarius* were investigated for their sensitivity against seven active compounds of synthetic fungicides. During the multi-year field trials, various novel management systems, including consortia of biocontrol agents, were revealed to be effective against Aspergillus sour rot and OTA production. However, expected variability was observed in the experimental results, indicating the dynamic character of biological systems and highlighting the possible inconsistency of PPPs’ efficacy in a changing environment. Furthermore, the IPM systems developed effectuated an optimized control of *A. carbonarius*, leading to 100% inhibition of OTA contamination, showing the importance of using both chemical and biological PPPs for disease management and prevention of fungal fungicide resistance. Finally, the majority of *A. carbonarius* tested strains were found to be sensitive against the pure active compounds used (fludioxonil, azoxystrobin, chlorothalonil, tebuconazole, cyprodinil, pyrimethanil and boscalid), with only a few exceptions of developed resistance towards boscalid.

## 1. Introduction

Ochratoxins are a group of toxic secondary metabolites produced by several fungal species. Ochratoxin A (OTA) is the most-studied mycotoxin in wine; thus, the European Commission (under regulation 2023/915) set a maximum tolerated level of 2 µg kg^−1^ for wines intended for human consumption [1]. Additionally, OTA is characterized as the most toxic member of the ochratoxin group and has been classified by the International Agency for Research on Cancer (IARC) as a class 2B carcinogen [2]. Balkan Endemic Nephropathy (BEN), a deadly kidney disease in humans, has been linked to OTA’s nephrotoxic effects [3]. Black Aspergilli are the primary cause of OTA contamination, as well as the cause of black rot in grape berries [4]. OTA is a mycotoxin produced by various fungi, primarily the genera *Aspergillus*, *Penicillium* and *Fusarium*, with notable species including *A. ochraceus*, *A. carbonarius*, *A. niger* and *P. verrucosum* [5]. However, the most significant OTA-producing species among black Aspergilli is *Aspergillus carbonarius* [6,7].

OTA presence in wine is largely due to the contamination of grapes while they are still in the vineyard, between veraison and pre-harvest or in the pre-winemaking process [8]. Vines and soil debris are the primary sources of inocula for *Aspergillus* species, which overwinter in vineyards [9]. Several ecophysiological studies that simulated real-world conditions have demonstrated that the growth of *A. carbonarius* as well as OTA production are influenced by environmental factors such as temperature, water availability and photoperiod [10]. The ideal temperature ranges for ochratoxigenic species’ development and OTA production in various substrates have been the subject of numerous investigations. However, the most favorable conditions for OTA biosynthesis can occasionally differ from those needed for fungal growth [11]. According to reports, *A. carbonarius*’ optimal growth temperatures range between 25 and 35 °C, whereas OTA production occurs between 20 and 25 °C [12]. In addition, excessive irrigation and rainfall before harvest contribute to an increase in the disease severity of *Aspergillus* spp., leading to berry splitting. Moreover, insect-induced wounds on berries create favorable conditions for black Aspergilli to enter [4]. Based on several studies, farming systems may also have an impact on *A. carbonarius* populations [13,14]. Kontaxakis and co-authors (2020) [13] discovered that organic vineyards had a higher incidence of *A. carbonarius* as compared to conventional fields. Contrariwise, the findings of Testempasis et al. (2022) [14] demonstrated that mycotoxigenic strains from conventional vineyards were isolated at higher rates.

OTA control strategies are primarily based on preventive measures (e.g., Good Agricultural Practices (GAPs), fungal-resistant or -tolerant crop varieties, correct timing of fungicide application and proper storage of vine commodities) [15]. The fungicides cyprodinil and fludioxonil, when combined, demonstrated the highest efficacy in reducing fungal growth and ochratoxin A (OTA) levels in grapes across multiple studies in France, Spain, Italy and Greece [16]. The study of Tjamos et al. [17] showed that in vineyards with low to intermediate *Aspergillus* infection, Switch^®^ (cyprodinil and fludioxonil) fungicide applications proved effective in reducing OTA-producing *Aspergillus* spp. and sour rot severity. Additionally, field trials have shown that other active ingredients, including pyrimethanil, fluazinam, iprodione and mepanipyrim, are also effective in reducing fungal growth in grapes [4]. Applying a mixture of cyprodinil (37.5%) and fludioxonil (25%) at veraison and 21 days before harvest successfully prevented black aspergilli in grapes in a field study conducted by Bellí and co-authors [18]. Chemical management is necessary to combat plant diseases, but because of the detrimental effects these chemicals have on the environment, human health and animal welfare, their usage is continuously being regulated by international legislation. In the last few decades, many chemicals used for the control of plant diseases have been outlawed, especially in EU nations [8].

At the same time, biocontrol agents seem to be a viable and less harmful way to control plant pathogens than conventional fungicides. In the case of *A. carbonarius* and *A. ochraceus*, studies have demonstrated the ability of yeast volatile compounds (VOCs) to suppress the development and reproduction of fungi or/and OTA production [19,20]. Bacteria belonging to *Bacillus* spp. have also demonstrated antifungal activity against *A. carbonarius*. More specifically, the application of *Bacillus subtilis* and *B. pumilus* resulted in a significant reduction in fungal growth on table grapes, which was attributed to the production of antibiotics such as iturin A by this bacterial species [21,22,23]. OTA production has also been found to be minimized in the presence of strains belonging to *Bacillus* spp. [24]. Furthermore, the inhibitory effects of different essential oils on black aspergilli and OTA have been explored by several studies, showing promising results in in vitro and in situ experiments [25,26]. From another perspective, the control of insects, especially *Lobesia botrana*, can contribute significantly to reducing the risk of OTA contamination [16,27,28]. Controlling the grape moth with the fungal biocontrol agent *Beauveria bassiana* has proved to reduce the risk of ochratoxin A contamination in grapes too [29].

While research has emphasized OTA detoxification strategies post-harvest, field control strategies for ochratoxin A during grape production remain largely understudied. Integrated pest management (IPM) systems are essential in vineyards to prevent the development of chemical resistance by the pathogens and ensure the long-term effectiveness of control strategies. The European Commission has established “The European Green Deal” action and the EU Directive 2009/128/EC on the sustainable use of pesticides and promotes the use of IPM strategies and alternative methods to control plant diseases and other pests. This research sought to shed light on the efficacy of synthetic fungicides and biological plant protection products (PPPs) in reducing OTA levels, as well as the development of IPM systems during a 4-year field trial, in two major Greek wine grape varieties, (cvs.) Malagousia and Savatiano.

## 2. Results

### 2.1. Evaluation of Plant Protection Products in Controlling Aspergillus Bunch Sour Rot in Vineyards

In the years 2016, 2017 and 2018, several biological and chemical PPPs were evaluated individually in experimental vineyards in order to assess the efficacy and consistency of the PPPs in controlling natural infections of Aspergillus sour rot and OTA biosynthesis across the years. Based on the results for the three years, different IPM strategies combing biological and chemical PPPs were implemented in the experimental vineyards in 2019.

#### 2.1.1. cv. Malagousia Disease Severity Assessment

**Figure 1:**
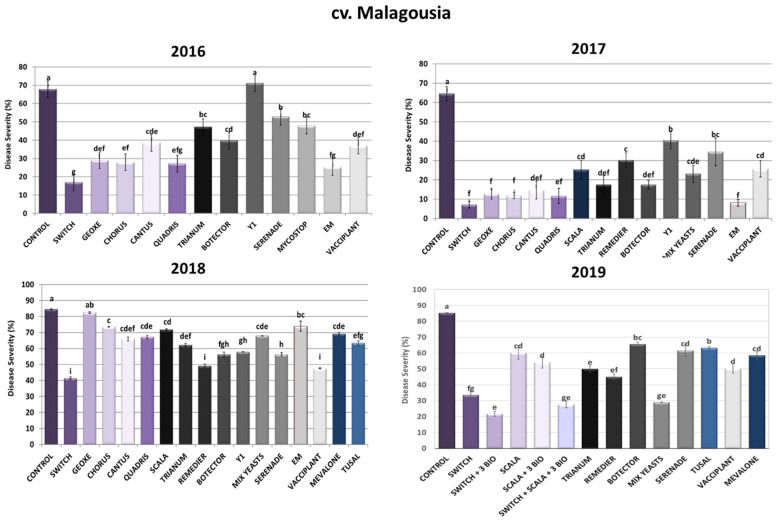
Disease severity of Aspergillus sour rot in grape berries of cv. Malagousia variety after PPP treatment of vines in the years 2016–2019. Statistical analysis was performed using one-way ANOVA followed by Tukey’s multiple-comparison post hoc test (*p* < 0.05). Letters above the graphs indicate differences between treatments.

As depicted in Figure 1, regarding the chemical control of *A. carbonarius* in cv. Malagousia vineyard plots, the synthetic fungicide Switch^®^, containing fludioxonil and cyprodinil, demonstrated the lowest percentages of disease severity during the years 2016 (75% reduction), 2017 (89% reduction), 2018 (51% reduction) and 2019 (66% inhibition). Accordingly, Chorus^®^, containing cyprodinil, reduced disease severity in high percentages, especially during the years 2016 (59% inhibition) and 2017 (82% inhibition). On the contrary, Geoxe^®^, containing fludioxonil, did not show consistent effectiveness across the three years of experiments, since it reduced disease severity up to 57%, 80% and 3% for 2016, 2017 and 2018, respectively. Cantus^®^ (boscalid) efficacy varied among the years of application, showing the lowest disease severity during 2017 (77% reduction), while in 2016 and 2018 it resulted in a reduction of symptoms at rates that did not exceed 43%. Similarly, Quadris^®^ (azoxystrobin) did not exhibit consistency in its efficacy, as in 2016 it effectuated a 60% reduction in disease severity caused by *A. carbonarius*, an 82% reduction in 2017 and a 21% reduction in 2018. Finally, Scala^®^, containing pyrimethanil, resulted in significantly less symptomatic rotten grape berries compared to the control in the experiment in 2017 (61% reduction), 2018 (15% reduction) and 2019 (29% reduction).

Concerning biological plant protection products (bioPPPs), ΕΜ^®^ (a mixture of beneficial microorganisms) demonstrated high reduction rates during 2016 (45%) and 2017 (87%), whereas in 2018 the reduction rate was found to be around 13%. Likewise, the application of the yeast *Aureobasidium pullulans*—Y1, isolated from the Laboratory of Plant Pathology, AUA, as an effective biocontrol agent against *A. carbonarius* and OTA production [30]—did not show any promising results during the three years of experiments on cv. Malagousia, since disease severity in the treated plants did not differ significantly from the control plants in 2016, and in the years 2017 and 2018 it inhibited disease progression at rates that did not surpass 37%. Contrariwise, Mix Yeasts, a consortium of five yeasts (*Candida railenensis*, *Aureobasidium pullulans* (two strains thereof)*, Rhodotorula mucilaginosa* and *Debaryomyces hansenii*) belonging to the beneficial microbe collection of the Laboratory of Plant Pathology, AUA, demonstrated low disease severity rates in 2017 (64% reduction) and 2019 (66% reduction). More specifically, for 2017, the inhibition rate was 64%, and for 2019 it was 66% (Figure 1). The individual strains comprising the Mix Yeasts previously demonstrated antagonistic activity against *A. carbonarius* and OTA (unpublished data).

The applications with the commercial bioPPP containing *Trichoderma harzianum* Trianum^®^ resulted in 30%, 73%, 27% and 41% sour rot reductions for 2016, 2017, 2018 and 2019, respectively. The efficacy of biopesticides containing microorganisms can vary from year to year due to environmental conditions (e.g., temperature, humidity, grape water stress, etc.) and fungal inoculum pressure from veraison till harvest. Furthermore, Remedier^®^ (*Trichoderma asperellum* and *Trichoderma gamsii*) sprayings effectuated 53%, 42% and 47% disease reductions for 2017, 2018 and 2019, respectively, showing a more constant efficacy for this product. As for Serenade^®^ (*Bacillus amyloliquefaciens*), disease severity differed significantly compared to the control plants, with decreases of 22% for 2016, 47% for 2017, 33% for 2018 and 28% for 2019. The other bacterial bioPPP, Mycostop^®^ (*Streptomyces griseoviridis*), showed a 63% decrease in severity during 2016. Vines treated with Botector^®^ (*Aureobasidium pullulans*) showed significantly fewer symptoms than plants on which no treatment was carried out, reaching inhibition rates of around 41%, 73%, 33% and 23% for 2016, 2017, 2018 and 2019, respectively. Tusal^®^, containing *Trichoderma* spp. strains, displayed an average sour rot reduction in both years tested between 25% and 26%, whereas Mevalone^®^, containing three essential oils, led to 18% and 31% disease severity reductions in 2018 and 2019, respectively. Finally, Vacciplant^®^ (laminarin) demonstrated a consistent reduction in disease severity symptoms, since rates were observed to be between 40 and 60% for all years tested (Figure 1).

During 2019, three Integrated Disease Management systems were developed and applied in cv. Malagousia vineyards to control sour rot based on the PPP efficacy during the previous three years of field experimentation. The fungicides Switch^®^ and Scala^®^ and the three bioPPPs (Serenade^®^, Trianum^®^ and Vacciplant^®^) were selected based on their efficacy in reducing *Aspergillus* sour rot and OTA production, as well as the different modes of action of their active ingredients. The combination of Switch^®^ with the bioPPPs Vacciplant^®^ (laminarin—a plant-resistance inducer), Serenade^®^ (*Bacillus amyloliquefaciens*) and Trianum^®^ (*Trichoderma harzianum*) resulted in 75% sour rot reduction, whereas Scala^®^ with the three bioPPPs led to a 36% disease decrease. When Switch^®^ and Scala^®^ were applied with the three bioPPPs, symptomatic berries were 67% fewer compared to the control vines. In 2019, the individual applications of these five PPPs led to disease reductions of 61% (Switch^®^), 29% (Scala^®^), 28% (Serenade^®^), 41% (Trianum^®^) and 41% (Vacciplant^®^). Interestingly, the Mix Yeasts was also effective in significantly reducing the disease severity by 66% in 2019. In conclusion, the IPM system combining Switch^®^ and three biological commercial products attained the optimum disease reduction in *A. carbonarius* (75%), highlighting the necessity of adopting such approaches to control sour rot in grapes (Figure 1).

#### 2.1.2. cv. Savatiano Disease Severity Assessment

**Figure 2:**
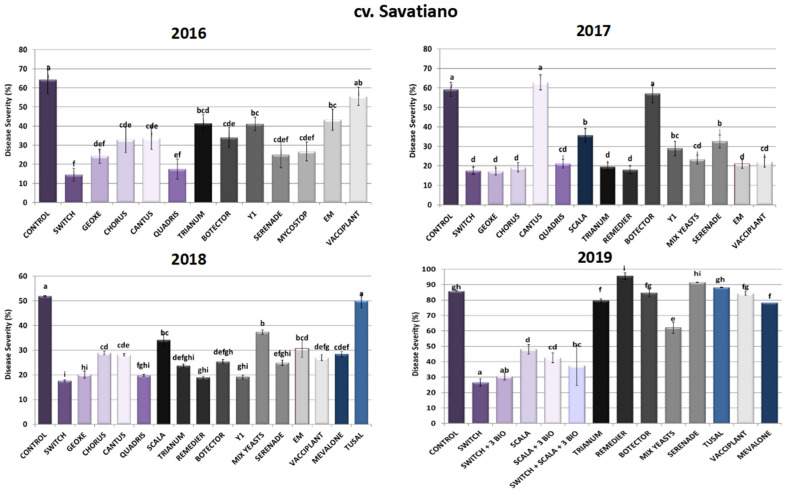
Disease severity of Aspergillus sour rot in grape berries of cv. Savatiano variety after PPP treatment of vines in the years 2016–2019. Statistical analysis was performed using one-way ANOVA followed by Tukey’s multiple-comparison post hoc test (*p* < 0.05). Letters above the graphs indicate differences between treatments.

As depicted in Figure 2, regarding the chemical control of sour rot in cv. Savatiano, fludioxonil and cyprodinil (Switch^®^) in combination proved to be the most efficient active compounds, following the pattern of the cv. Malagousia results, reaching inhibition rates from 66% up to 78%. Accordingly, Geoxe^®^ (fludioxonil), Chorus^®^ (cyprodinil) and Quadris^®^ (azoxystrobin) demonstrated significant reductions in disease severity (44–73%) in all three years tested, indicating a possible chemical approach to managing the disease caused by *A. carbonarius* by rotating the efficient chemical formulations to avoid the development of fungal resistance to specific active substances. Conversely, boscalid’s (Cantus^®^) efficacy varied among the years of application, as observed in cv. Malagousia, decreasing disease severity at a rate of around 40% in the years 2016 and 2018, while in 2017 Cantus^®^-treated plants demonstrated symptoms at rates observed in the control plants as well.

As for the biological formulations, Serenade^®^ (*B. amyloliquefaciens*) substantially decreased disease severity (45–61%) in the first three years of the experiments (2016, 2017 and 2018), with a differentiation of results during 2019, when it did not show any significant inhibition of the disease caused by *A. carbonarius*. This may have been due to the unfavorable environmental conditions for the establishment and the growth of the beneficial microorganism included in the specific bioPPP. There was variation in the results for Vacciplant^®^ (laminarin) for the years tested, with 13% inhibition of the disease in 2016, 63% in 2017, 48% in 2018 and 2% in 2019. During field experiments in 2019, when Vacciplant^®^ was combined with other bioPPPs and chemicals in the context of an IPM program, the results proved to be promising, according with the observations made during the cv. Malagousia field trials for the same treatments. Furthermore, *Trichoderma harzianum* (Trianum^®^) applications showed reductions in the numbers of symptomatic berries at rates between 36 and 67% compared to the control experiments, during the first three years of field trials in cv. Savatiano. Contrariwise, 2019 Trianum^®^ sprays did not result in any significant reduction in disease severity, though when combined with the other PPPs tested in an integrated approach, the findings were promising. Moreover, Remedier^®^ (*Trichoderma asperellum* and *Trichoderma gamsii*) exhibited high reduction rates in 2017 (70%) and 2018 (64%), while in 2019 plants treated with the specific PPP did not show any decrease in symptoms caused by *A. carbonarius*. The bioPPP Mycostop^®^ (*Streptomyces griseoviridis*) also showed a significant reduction (59%) in severity in cv. Savatiano, as in cv. Malagousia, in 2016 (Figure 2).

On the other hand, the outcome of Botector^®^ (*Aureobasidium pullulans*) applications in cv. Savatiano vineyards displayed great alterations of disease severity rates during all years of experimentation. More specifically, in 2016, Botector^®^ inhibited the progress of sour rot in percentages between 47% and 51% in 2018, whereas in 2017 and 2019 the percentages of diseased berries did not vary significantly from the rates of grapes harvested from the control plants. Concerning the corresponding non-commercial isolate of *Aureobasidium pullulans* (Y1), disease severity levels were found to be lower than the ones calculated for Botector^®^ treatments in each year of the experiments in cv. Savatiano, indicating the possibility of Y1 being a better adapted yeast strain with respect to the local conditions in Greek vineyards. These results suggest a further investigation aiming at the potent development of a formulation of the specific isolate for the control of sour rot in Greek vineyards. Likewise, Mix Yeasts inhibited *A. carbonarius* disease severity by 61% in 2017, 28% in 2018 and 28% in 2019. Additionally, EM^®^ effectuated some promising results by demonstrating a 33% disease severity reduction during 2016, a 65% decrease in 2017 and a 41% inhibition of disease during 2018. Finally, Mevalone^®^ and Tusal^®^ showed 45% and 4% sour rot reductions in 2018, respectively, while in 2019 they did not alter the symptoms in plants treated with these two formulations compared to the control of the experiment (Figure 2).

Regarding the evaluation of the IPM strategies to control grape sour rot in 2019 in cv. Savatiano vineyards, the results showed that the combination of Switch^®^ with the bioPPPs Vacciplant^®^, Serenade^®^ and Trianum^®^ resulted in 65% disease severity reduction, whereas Scala^®^ with the three bioPPPs led to 50% severity decrease. When Switch^®^ and Scala^®^ were applied with the three bioPPPs, the inhibition of the disease was 56% compared to control vines. In 2019, the individual applications of these five PPPs led to disease reductions of 69% (Switch^®^), 44% (Scala^®^), 0% (Serenade^®^), 7% (Trianum^®^) and 2% (Vacciplant^®^), indicating strongly that the efficacy of biological products depends on the variety, the environmental conditions and other factors in vineyards (Figure 2).

#### 2.1.3. cv. Malagousia—Ochratoxin A Levels

**Figure 3:**
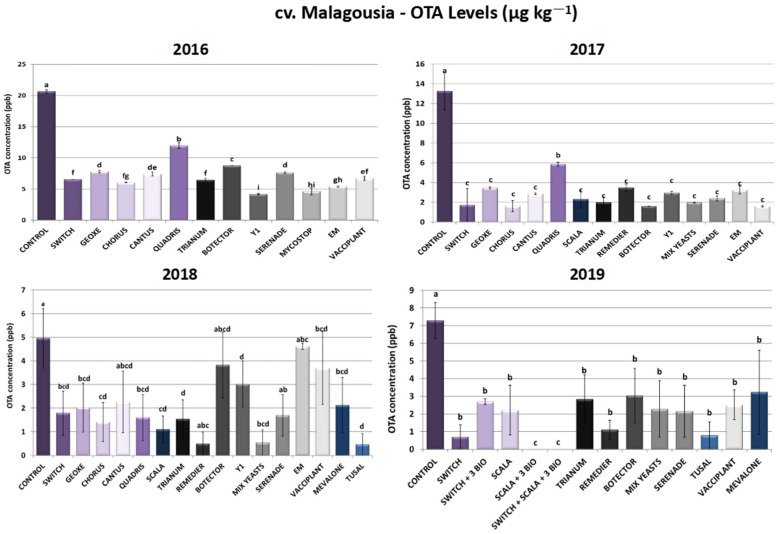
Ochratoxin A levels (μg kg^−1^) produced by *Aspergillus carbonarius* in cv. Malagousia after PPP treatment of vines in the years 2016–2019. Statistical analysis was performed using one-way ANOVA followed by Tukey’s multiple-comparison post hoc test (*p* < 0.05). Letters above the graphs indicate differences between treatments.

As depicted in Figure 3, all treatments managed to lower ochratoxin A in levels that varied significantly compared to untreated control plants in cv. Malagousia. During 2016, Switch^®^ and Chorus^®^ applications led to 68% and 71% reductions in OTA levels—outcomes that are consistent with the decrease in disease severity due to these two chemical fungicides in the current year of field trials. The following years, the combination of fludioxonil and cyprodinil (Switch^®^), as well as cyprodinil as a sole active ingredient in Chorus^®^ treatments, inhibited OTA biosynthesis at rates above 87% (2017) and above 64% (2018), while in 2019 Switch^®^ sprayings resulted in a 90% reduction in OTA produced by *A. carbonarius*. The OTA reduction rates are in accordance with the decrease in disease severity in plants sprayed with Switch^®^ and Chorus^®^ in the respective years of field trials. Accordingly, Geoxe^®^ (fludioxonil) effectuated a high decrease in OTA levels in a range between 60 and 74% from 2016 to 2018, while it did not demonstrate consistency in disease severity reduction across the years (3–80% reduction). In like manner, Cantus^®^, inhibition rates in cv. Malagousia vineyards were calculated to be above 54% and up to 78% for 2016, 2017 and 2018. Contrariwise, Quadris^®^ (azoxystrobin) demonstrated the lowest percentages of OTA inhibition compared to the other synthetic PPPs tested, but still its applications resulted in 42%, 56% and 68% inhibition in 2016, 2017 and 2018, respectively. Finally, concerning chemical formulations, Scala^®^, containing pyrimethanil as an active compound, displayed inhibition rates of 83%, 78% and 70% for 2017, 2018 and 2019, while regarding disease severity reduction, rates were calculated to be particularly lower (15–61%). Notably, when pyrimethanil (Scala^®^) was combined with the bioPPPs Vacciplant^®^, Serenade^®^ and Trianum^®^ in the IPM scheme developed during 2019, OTA reduction rates reached 100%. However, disease reduction rates reached 67%, indicating the possibility of alternative modes of action in minimizing OTA levels.

Regarding the efficacy of yeast biocontrol agents in cv. Malagousia, Mix Yeasts (*Candida railenensis*, *A. pullulans*, *Rhodotorula mucilaginosa* and *Debaryomyces hansenii*) demonstrated the highest decrease in OTA levels compared to the commercial bioPPP Botector^®^ (*A. pullulans*), followed by the non-commercial strain Y1 of *A. pullulans.* More specifically, Mix Yeasts applications led to an 85% reduction in OTA levels in 2017, an 89% reduction in 2018 and a 69% reduction in 2019, while disease severity rates ranged between 60 and 65% during 2017 and 2019. The treatments with the isolate Y1 resulted in an OTA decrease of 80% during 2016 and a reduction of 78% in 2017, whereas in 2018 inhibition rates did not exceed 40%. Interestingly, while *A. pullulans* strain Y1 did not demonstrate a significant reduction in disease severity, its ability to minimize ochratoxin A produced by the fungus was pronounced, suggesting potentially multifaceted mechanisms of action and not a direct antagonism against *A. carbonarius*. Yeasts may have a direct inhibitory effect on mycotoxin production independently of their growth-suppressing effect [31]. Yeast cells and their extracts have often been reported as a means of OTA detoxification [32]. Finally, Botector^®^ demonstrated significantly lower levels of OTA levels compared to control plants during the 4-year field trials that were performed—an observation that was found to be in accordance with disease severity results, since the same bioPPP resulted in significantly fewer symptoms in treated vines (Figure 3).

Serenade^®^ (*Bacillus amyloliquefaciens*) sprayings resulted in 63%, 82%, 66% and 70% inhibition of OTA biosynthesis by the fungus in the years 2016, 2017, 2018 and 2019, respectively, even though disease severity percentages were substantially elevated, especially during 2016 and 2018. This could support the hypothesis that there are other indirect modes of action underlying the disease reduction, since bacteria belonging to *Bacillus* spp. reportedly also contribute to the detoxification of various toxins, including OTA [33,34,35]. The other bacterial bioPPP, Mycostop^®^, also led to a significant 78% reduction in OTA biosynthesis in 2016. Trianum^®^, Remedier^®^ and Tusal^®^, containing fungi belonging to *Trichoderma* spp., showed promising results with inhibition levels from 60% to 91% for all years of field trials in cv. Malagousia vineyards, with the latter reaching the highest decrease levels (91%). Remedier^®^ and Tusal^®^, containing two different species of *Trichoderma* spp., may have a higher efficacy in reducing OTA levels compared to Trianum^®^, which contains only one species of the genus, due to the potent alternative mechanisms of action of the products’ formulations. In Vacciplant^®^-treated plants, variation in OTA levels was observed, with inhibition rates ranging from 65% to 88% in the years 2016, 2017 and 2019 and a rate of 25% in 2018, whereas disease severity reduction was consistent across the years of applications, indicating that the pathogen’s biomass reduction is not always analogous to the reduction in the produced OTA. Correspondingly, EM results were not consistent across the years tested, with calculated inhibition rates between 74 and 75% in 2016 and 2017 and a rate of 7% in 2018 (Figure 3). The combination of the chemicals Scala^®^ and Switch^®^ with the three biopesticides (Serenade^®^, Trianum^®^ and Vacciplant^®^) led to 100% mitigation of OTA. The other two tested IPM schemes, Switch^®^ + 3 bioPPPs and Scala^®^ + 3 bioPPPs, resulted in 63% and 100% OTA reductions, respectively (Figure 3).

#### 2.1.4. cv. Savatiano—Ochratoxin A Levels

**Figure 4:**
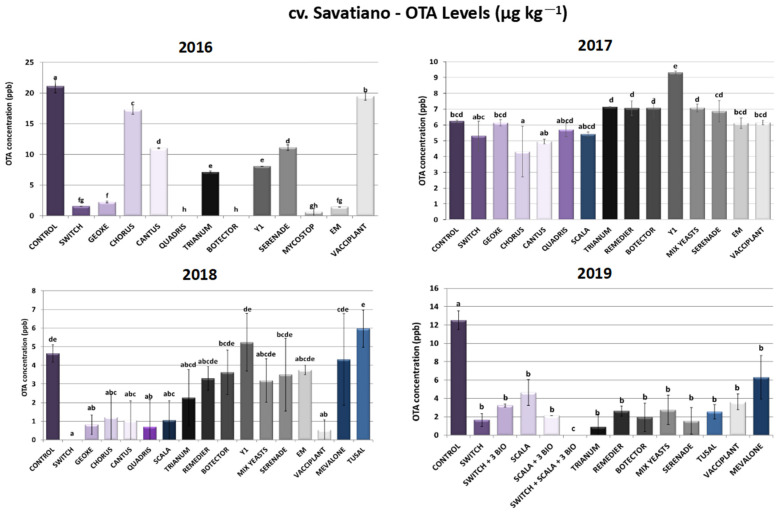
Ochratoxin A levels (μg kg^−1^) produced by *Aspergillus carbonarius* in cv. Savatiano after PPP treatment of vines in the years 2016–2019. Statistical analysis was performed using one-way ANOVA followed by Tukey’s multiple-comparison post hoc test (*p* < 0.05). Letters above the graphs indicate differences between treatments.

As presented in Figure 4, significant variability in results was observed across the trial years in cv. Savatiano, indicating potential influences of environmental factors, the application timing of PPPs and/or the specific grape variety. During 2016, Quadris^®^-treated plants demonstrated the lowest OTA levels, reaching 100% inhibition of its biosynthesis. Nevertheless, disease severity reduction rates did not exceed 73%. Concerning the other chemical fungicides, Switch^®^ and Geoxe^®^ resulted in a decrease above 90%, followed by Cantus^®^ and Chorus^®^ with 48% and 18% reductions, respectively. Switch^®^, among all the chemical formulations tested, showed the highest decrease in sour rot symptoms. As for the biological control of sour rot during the year 2016, Botector^®^ and EM^®^ outperformed the other bioPPPs, as they effectuated a decrease in OTA levels above 93%, with the former demonstrating 100% inhibition of ochratoxin A produced by *A. carbonarius*. Trianum^®^ and *A. pullulans* strain Y1 followed, with reduction rates ranging between 62 and 66%. Serenade^®^ and Mycostop^®^ sprayings led to 47% and 78% inhibition rates of ochratoxin A contamination, respectively, in 2016, while Vacciplant^®^ did not show any significant alteration in OTA levels in the same year.

Conversely, in 2017, the range of biocontrol agents tested did not display any significant reduction in OTA levels. However, the efficacy of the synthetic plant protection products was lower, with the Scala^®^, Switch^®^, Chorus^®^ and Cantus^®^ inhibition rates ranging between 13 and 31%, while Geoxe^®^ and Quadris^®^ did not demonstrate any notable reduction (Figure 4).

During 2018, all the chemical fungicides reached OTA inhibition rates that exceeded 74%, with Switch^®^ applications leading to a 100% reduction in OTA, since contamination levels were found to be under the limit of detection of the quantification method (ELISA). On the other hand, the results among the bioPPPs tested showed several variations. Vacciplant^®^, which in 2016 did not alter OTA levels compared to the untreated plants, demonstrated the highest inhibition (88%) compared to all the other bioPPPs that inhibited OTA at rates that did not surpass 50%. The variable efficacy of PPPs, especially those of microbial origin, highlights the need for ongoing research into environmental or genetic (cultivar) factors that influence their effectiveness (Figure 4).

Furthermore, during the last year of experiments (2019), the range of bioPPPs tested showed a high inhibition of contamination levels, with reduction rates ranging from 50 to 93%. Trianum^®^, Botector^®^, Serenade^®^ and Tusal^®^ applications in cv. Savatiano vineyards led to a decrease in OTA levels above 80%. It is of sufficient importance to highlight that during 2019 the disease severity rates in Botector^®^-treated plants did not vary significantly from the rates of untreated plants (controls), despite the reduction in OTA. Likewise, Serenade did not manage to reduce disease severity, despite the decrease in OTA produced by *A. carbonarius*. Synthetic fungicides and their combination with biological PPPs in the context of IPM systems developed during 2019 gave the optimum results. The combination of two chemicals (Scala^®^ and Switch^®^) and three biologicals (Serenade^®^, Trianum^®^ and Vacciplant^®^) led to 100% minimization of OTA, with levels found below the LOD of the ELISA method. The other two tested IPM schemes, Switch^®^ + 3 bioPPPs and Scala^®^ + 3 bioPPPs, showed 74% and 83% OTA reductions, respectively (Figure 4).

### 2.2. In Vitro Assessment of A. carbonarius’ Sensitivity to Fungicides

Nine Greek isolates of *A. carbonarius* were examined for their potential to develop resistance to seven active substances of commercial fungicides used in viticulture, including the ones that were evaluated in the previously described vineyard studies (Table 1). The active substances azoxystrobin and boscalid were evaluated for their ability to inhibit conidial germination of *A. carbonarius* isolates. The results from this experiment revealed a diverse range of sensitivities to conidial germination inhibition. Regarding azoxystrobin, almost all isolates exhibited EC_50_ values below 0.1 μg mL^−1^. More specifically, two isolates demonstrated EC_50_ values below 0.025 μg mL^−1^, four isolates showed EC_50_ values below 0.1 μg mL^−1^ and only one isolate displayed an EC_50_ value slightly above 0.1 μg mL^−1^, indicating a relative sensitivity of the fungus towards this active substance. For boscalid, the results varied, as three out of the nine isolates were identified as resistant to this active substance. Specifically, strain EASN14 exhibited an EC_50_ value greater than 5 μg mL^−1^, strain AC33 demonstrated an EC_50_ value greater than 3 μg mL^−1^ and strain AC55 showed an EC_50_ value greater than 2.5 μg mL^−1^. For the remaining six isolates, EC_50_ values were calculated to be below 0.2 μg mL^−1^, with the strain AC29 being the most sensitive to boscalid (EC_50_ < 0.1 μg mL^−1^).

Regarding the active substances fludioxonil, cyprodinil, pyrimethanil, tebuconazole and chlorothalonil, which were tested for their ability to inhibit mycelial growth of the nine highly ochratoxigenic *A. carbonarius* isolates, EC_50_ values showed significant variations. Interestingly, the results for fludioxonil and cyprodinil were found to be equivalent. For fludioxonil, all isolates exhibited high sensitivity, with EC_50_ values below 0.1 μg mL^−1^—the minimum concentration tested. Accordingly, all isolates tested on plates containing cyprodinil were characterized as sensitive, with EC_50_ values below 0.01 μg mL^−1^. Moreover, seven out of nine isolates were proved to be sensitive to pyrimethanil, with EC_50_ values below 0.05 μg mL^−1^, while the EC_50_ value for isolate DASN43 was determined to be 0.08 μg mL^−1^ and that for EASN14 was determined to be 0.1862 μg mL^−1^, which was the highest value calculated towards the active compound. As for tebuconazole, all isolates were characterized as sensitive, with EC_50_ values below 0.8 μg mL^−1^. However, for chlorothalonil, one isolate, BASN31, had an EC_50_ value above 1 μg mL^−1^. The rest of the strains were characterized as sensitive, with EC_50_ values below 0.6 μg mL^−1^. The results of these experiments are presented in detail in Table 1.

## 3. Discussion

While previous research has investigated individual approaches to managing *A. carbonarius* and OTA in vineyards, there is a lack of further information on the effectiveness of available commercial biological and chemical PPPs and on biocontrol agents or integrated management strategies under development in pilot vineyards. This study aimed to address this gap by conducting a multi-year, large-scale field trial in two important Greek wine white varieties, cv. Malagousia (early variety) and cv. Savatiano (late variety), in vineyards with natural *A. carbonarius* infections. Chemical plant protection products (PPPs), as well as biological non-commercial and commercial PPPs, were evaluated by their application as single treatments or/and in combinations in the context of IPM systems. The variability observed in the experimental results was expected and is likely attributable to the high complexity of the systems under investigation. Biological systems are mostly dynamic and influenced by a plethora of factors, including genetic host and fungal variability, environmental fluctuations, harvest periods, and other extrinsic aspects. Subsequently, different *A. carbonarius* isolates were used for in vitro sensitivity experiments on pure active substances included in the synthetic fungicides tested in the vineyards.

Regarding chemical control, cyprodinil (Chorus^®^ and Switch^®^), fludioxonil (Geoxe^®^ and Switch^®^), boscalid (Cantus^®^), azoxystrobin (Quadris^®^), pyrimethanil (Scala^®^), chlorothalonil and tebuconazole—registered fungicides for use in vines—were selected to be evaluated for *A. carbonarius* management and resistance development. The most successful approach reported by Bellí and colleagues [18] in preventing black aspergilli in grapes was a combination of cyprodinil (37.5%) and fludioxonil (25%) applied twice: at veraison and again 21 days before harvest. Another effective treatment was a single application of this mixture at veraison, followed by a cyprodinil (50%) application 21 days before harvest. Likewise, Switch^®^ fungicide applications, particularly in vineyards with low to moderate Aspergillus infection, effectively reduced the prevalence of OTA-producing Aspergillus species and limited the severity of sour rot [17]. Moreover, Chorus^®^, containing cyprodinil, proved most effective in significantly reducing ochratoxin A (OTA) accumulation in grapes during dehydration [36]. On the contrary, the study of Thomidis et al. [37] reported that pre-harvest applications of Switch^®^ (cyprodinil + fludioxonil) 62.5 WG and Switch^®^ 62.5 WG + Rhapynal^®^ (a biosurfactant based on rhamnolipids) proved effective in lowering black *Aspergillus* spp. populations but had no discernible impact on OTA contamination in grapes. The observations of the current study highlighted the ability of cyprodinil and fludioxonil to reduce disease severity and OTA production by *A. carbonarius* at high rates (even 100%) during the 4-year field trial in both cv. Malagousia and cv. Savatiano vineyards. Additionally, in vitro tests revealed the sensitivity of *A. carbonarius* strains isolated from various areas around Greece towards cyprodinil and fludioxonil, since most of the isolates’ EC_50_ values were calculated as being below the minimum concentrations tested for each active compound, which were 0.01 μg mL^−1^ and 0.1 μg mL^−1^, respectively, indicating that none of the tested isolates developed resistance to these fungicides.

Furthermore, the accumulation of OTA in grapes can be effectively managed through the use of fungicides, including azoxystrobin, as evidenced by studies conducted by Bellí et al. (2006) [38] and Lo Curto et al. (2004) [39]. Quadris^®^, containing azoxystrobin, which was used in the field trials, did not display consistent efficacy across the years tested—a fact that could be explained by the variations in environmental conditions, the grape harvest times or/and the pathogen inoculum levels in the vineyards, since the majority of *A. carbonarius* isolates demonstrated relative sensitivity towards azoxystrobin, showing EC_50_ values below the lower dose tested (0.025 μg mL^−1^). These findings have been confirmed by other researchers [39], who reported a 96.5% reduction in OTA levels when azoxystrobin was applied.

On the other hand, particular *A. carbonarius* strains (EASN14, AC33 and AC55) cultivated on boscalid-modified plates demonstrated relative resistance, supporting the observations of other studies indicating the insensitivity of this *Aspergillus* species to this active compound. Indeed, the efficacy of Cantus^®^ (boscalid) in reducing sour rot’s disease severity varied among the years, and its rates of efficacy in inhibiting OTA biosynthesis were found to be low. Boscalid-resistance-conferring mutations in the SdhB subunit of respiratory complex II are very common mechanisms of developing resistance in several mycotoxigenic fungi [40,41,42]; thus, the alteration and combination of this active substance with other chemical compounds with different modes of action is crucial to avoid the development of resistance by *A. carbonarius*.

Scala^®^ (pyrimethanil) applications resulted in observations that differed among the two varieties used in the current study, as OTA levels in cv. Malagousia decreased in high percentages during the years of the field trials, while in cv. Savatiano pyrimethanil did not inhibit OTA levels at significant rates. This could be explained by the divergent metabolic pathways of each variety in relation to Scala^®^ and OTA production—a complex that warrants further investigation. Contrariwise, a study by Zouhair et al. (2014) [43] revealed that applying sub-lethal doses of certain fungicides, including pyrimethanil, to *A. carbonarius* and *A. niger* resulted in reduced fungal growth rates, while, at the same time, it increased production of the mycotoxin OTA.

Moreover, chlorothalonil and tebuconazole were used in the in vitro tests with respect to the sensitivity of several *A. carbonarius* isolates towards certain active compounds. More specifically, almost all fungal strains showed relative sensitivity towards chlorothalonil, with EC_50_ values slightly below 0.05 μg mL^−1^. In like manner, while all fungicides used in the study of Terra et al. (2016) [44] reduced fungal growth of *A. carbonarius*, chlorothalonil (82.5%) proved most effective, completely inhibiting growth at all tested doses. Accordingly, EC_50_ values regarding tebuconazole were found to be between 0.2 and 0.5 μg mL^−1^—results that are reinforced by another study in which tebuconazole at the lowest doses (<0.25 μg mL^−1^ x the dose suggested by the manufacturer) showed the highest increase in OTA production in a grape medium [38].

Regarding biological control of Aspergillus bunch sour rot, research papers have shown promising results for *Aureobasidium pullulans* yeasts in mitigating *A. carbonarius* on grapes [45]. Studies have demonstrated that *A. pullulans* can effectively reduce the severity of disease caused by *A. carbonarius*, leading to a decrease in the production of OTA as well. This reduction in OTA levels is attributed to the antagonistic interactions between the pathogen and the beneficial microorganism, where *A. pullulans* competes with *A. carbonarius* for resources and, in addition, it produces compounds that inhibit OTA biosynthesis [30,46]. Our study highlights the potential of this species to manage *A. carbonarius* infections in vineyards—an observation made through the application of commercially available and non-commercial, under-development *A. pullulans* biocontrol strains. More specifically, *A. pullulans* strain Y1, with proven efficiency against *A. carbonarius* and OTA reduction [30], even though in cv. Malagousia it did not show any significant inhibition in disease severity, OTA biosynthesis was inhibited at high rates, possibly indicating other underlying mechanisms of action than direct antagonism for space. Yeasts may have a direct inhibitory effect on mycotoxin production, independently of their growth-suppressing effect [31], and yeast cells and their extracts are often reported as means of OTA detoxification [32]. In cv. Savatiano, the results were still promising, since Y1 applications resulted in a disease reduction higher than those observed for treatments with other chemical and biological plant protection products on the market, followed by a significant reduction in OTA contamination as well. These findings confirm the results obtained from previously implemented field trials on the Greek island of Rhodes (Greece) and in Corinthos County (Greece), where the biocontrol agent Y1 (*A. pullulans*) was evaluated on two red grape varieties: cv. Grenache Rouge and cv. Agiorgitiko. The results of this study showed that Y1 was equally effective as the commercial fungicide fludioxonil + cyprodinil (Switch^®^) in reducing sour rot infection, *A. carbonarius* presence on grapes at harvest and OTA contamination of grape juice [30]. Moreover, Mix Yeasts, a consortium of five yeasts (*Candida railenensis, Aureobasidium pullulans* (two strains), *Rhodotorula mucilaginosa* and *Debaryomyces hansenii*) demonstrated low disease severity rates in both grape varieties and a high decrease in OTA levels in cv. Malagouzia, implying a promising formulation to be developed as a new biopesticide. As for Botector^®^, its sprayings led to diverse results from year to year in both disease severity experiments and OTA quantification analyses.

Biocontrol agents belonging to *Trichoderma* spp. are a popular category of bioPPPs due to their multiple modes of action, such as fungal parasitism and antagonism for space and nutrients, as well as the induction of plant defense genes [47,48]. Hence, three biological control products (Trianum^®^, Tusal^®^ and Remedier^®^) containing a single or multiple strains belonging to this genus were evaluated. Notably, the inhibition of disease severity was not analogous to OTA inhibition rates, since, for example, Remedier^®^ did not significantly reduce disease severity in cv. Savatiano but reached an inhibition of OTA biosynthesis that exceeded 80%. However, Remedier^®^ and Tusal^®^, containing more than one species of *Trichoderma*, showed a higher efficacy than Trianum^®^ (containing only one strain of *T. harzianum*). A mixture of species may be better suited to adapting to varying environmental conditions and host genotypes, and this can result in more consistent performance across different years of application. Additionally, multiple species may also promote better plant–microbe interactions and alter the microbiomes of grape leaves and berries’ surfaces, potentially leading to improved resilience against biotic or/and abiotic stressors, which can further enhance the overall efficacy of the treatment. In a though-provoking study, it was proved that when *A. carbonarius* was co-cultured with strains of *A. niger, Trichoderma* spp., and strains of *Cladosporium* spp., *Acremonium* spp. and *Geotrichum* spp., OTA synthesis was completely inhibited, indicating the importance of fungal–fungal interactions in complex pathosystems. These findings showed that the presence of various non-toxic fungal strains growing together in paired cultures had a substantial impact on the amount of OTA produced by the *A. carbonarius* strain [49]. Similar studies indicate that bacterial–fungal interactions influence mycotoxin production, in addition to environmental factors, with multiple reports of detoxification of mycotoxins in the literature. The study of Venkatesh and Keller (2019) presents several examples of degradation of Fusarium mycotoxins mediated by microbes, plants and insects [50].

The biocontrol product Serenade^®^, containing *B. amyloliquefaciens* (formerly *B. subtilis*), has proved effective in controlling brown rot in grapes caused by *Monilinia* spp. [51]. Studies indicate the ability of *Bacillus* spp. to reduce OTA in grapes, such as the study of Diogo-Silveira et al. (2021), where it was reported that *B. velezensis* was the most promising among various *Bacillus* spp. strains for completely inhibiting fungal growth and production of all ochratoxins [24]. Serenade^®^, when used in cv. Malagousia and cv. Savatiano vineyards during 2016, 2017, 2018 and 2019, did nοt significantly reduce disease severity by *A. carbonarius* but resulted in high rates of inhibition of OTA biosynthesis—a pattern that was observed in almost all biological treatments due to the possible indirect mechanisms of action they possess against plant pathogens. Beneficial microbes might effectively reduce OTA production in specific niches or at certain growth stages of *A. carbonarius*. However, this reduction might not be sufficient to prevent disease establishment or progression if the fungus has already colonized the host tissue and initiated the disease process. Further research is needed to fully elucidate these complex interactions.

Until now, studies have shown that essential oils can reduce OTA production by reducing fungal growth or/and conidia production by fungi [52,53]. Mevalone^®^ consists of geraniol, thymol and carvacrol—substances reported to have a detoxification effect on OTA produced by *Aspergillus* spp. [53,54,55]. The sprayings with this bioPPP in vineyards did not effectuate any noteworthy reduction in disease severity or in OTA levels compared to all other treatments. Likewise, Vacciplant^®^ treatments resulted in various results among the years and varieties, possibly because this product has been characterized as a plant biostimulant; thus, its main mode of action is the induction of plant defenses. *A. carbonarius* thrives in warm temperatures, particularly in the range of 25–35 °C, whereas OTA production occurs between 20 and 25 °C [12]. If temperatures are too low, fungal growth slows and OTA contamination becomes less likely. High temperatures can also stress grapevines and make them more susceptible to fungal infections, particularly during ripening, when the grape berries are more vulnerable. Additionally, excessive heat can increase grape ripeness and soften the berries, creating a more favorable environment for pathogen infection and the rapid onset of sour rot. In general, environmental conditions such as air temperature, relative humidity and berry wetness, as well as other factors such as grape variety, diseases, pests, the nutrient status of vines, plant abiotic stress and soil conditions, can play crucial roles in OTA contamination and *Aspergillus* berry rot disease in vines. The implementation of a large, multivariate and multiyear study in different geographical locations needs to be considered in the future in order to investigate the correlation of *Aspergillus* growth, OTA contamination and several other environmental, cultural, soil and plant physiological factors.

The effectiveness of biocontrol agents (BCAs) is influenced by a complex interplay of factors, including the concentration of the BCA itself, the pathogen’s inoculum, and the host plant species and variety. Unfavorable environmental conditions (temperature, humidity, etc.), as well as plant-related factors, are crucial, as they impact the establishment, growth and survival of both the BCA and the pathogen and can alter the physiological and metabolic processes of all three players in this interaction. If the conditions in the field are not optimal for the biocontrol agent, it may not function effectively. Some BCAs may even become less viable or fail to thrive under stress, which can lead to poor performance. High temperatures and heat waves in a field can significantly influence the population levels and the mode of action of BCAs [56]. Sometimes, biocontrol agents may also unexpectedly lead to increased disease severity in the field due to several factors. BCAs compete with native microbes for nutrients, space and other resources. In some cases, if the biocontrol agent is less competitive than other organisms, e.g., under extreme environmental conditions, it may fail to establish, allowing the harmful pathogen to proliferate more than it would have in the absence of the biocontrol agent. BCAs may also produce secondary metabolites or enzymes that are harmful to plants in certain environmental conditions, or they may create an imbalance in microbial communities that leads to increased disease pressure. The effectiveness of biocontrol agents depends also on proper application techniques. If the agent is applied incorrectly (e.g., at the non-optimum plant growth stage, at the wrong time, in the wrong concentration or using unsuitable methods), it may not establish or function as expected [56]. Finally, the development of IPM systems during 2019 led to optimized results and to a sound solution to the selection of an effective control strategy, since inhibition of both disease severity and OTA levels reached the highest rates when Scala^®^, Switch^®^ and the three bioPPPs Vacciplant^®^ (laminarin: a plant-resistance inducer), Serenade^®^ (*Bacillus amyloliquefaciens*: a bacterium with antagonistic activity due to production of antibiotics such as iturin A) and Trianum^®^ (*Trichoderma harzianum:* a fungus that controls pathogenicity through different mechanisms, including mycoparasitism, secretion of secondary metabolites, antibiotic secretion, competition for nutrients and space, and plant resistance induction) were combined.

By combining chemical treatments with biological control agents with diverse modes of action, our experiments demonstrated that this synergistic strategy yields optimal results in the management of sour rot and OTA. The integration of various control methods allows for more effective suppression of *A. carbonarius*, as proved in the current study, while simultaneously addressing the risk of OTA contamination. This multifaceted approach not only improves grape quality and yield, but also supports sustainable viticulture practices, ensuring the long-term health of vineyard ecosystems.

## 4. Conclusions

Overall, the findings of this study highlight the potential of both chemical and biological PPPs to serve as effective tools for the control of grape bunch sour rot and OTA biosynthesis within an integrated pest management (IPM) framework in commercial vineyards. The effectiveness of several commercial biological PPPs and under-development biocontrol agents in the management of *A. carbonarius* and OTA biosynthesis was discovered for the first time in pilot vineyards. The results of this study also showed that consortia of biocontrol species may be better suited to adapting to varying environmental conditions and host genotypes and that this can result in more consistent performance across different years of application. Combining PPPs with various mechanisms of action against *A. carbonarius* and OTA biosynthesis will enable grape producers to improve their practice, resulting in wine production systems that are more robust and sustainable. To our knowledge, this is the first report of a large-scale, multi-year evaluation of biological and chemical PPPs and the development of efficient IPM systems in controlling sour rot and OTA in vineyards.

## 5. Materials and Methods

### 5.1. Field Trials

This study was carried out in two commercial vineyards of cv. Malagousia (an early white wine variety that is harvested mid- to late August) and cv. Savatiano (a late white wine variety that is harvested early to mid-September) belonging to the Agricultural University of Athens (AUA), Greece, during the years 2016, 2017, 2018 and 2019. The vineyards followed standard agronomic and cultivation practices for the region, employing irrigation. The vines were trained to grow vertically in a double cordon system—a method known as vertical shoot position (VSP). Additionally, they received a normal fertilization program in winter/spring and were pruned during the spring and summer periods.

#### 5.1.1. Experimental Design—Plant Protection Products

The experimental set up in vineyards consisted of completely randomized blocks, with 30 replicates (single plants) for each treatment. The synthetic and biological PPPs and the biocontrol agents used in the current study are presented in Table 2 and Table 3, respectively. PPPs were sprayed using knapsack sprayers at the maximum recommended certified doses of the manufacturers. For the single applications of chemical or biological PPPs, the products were applied twice; the first spraying was performed when the grapes of each variety were changing color (veraison), in mid-July for cv. Malagousia and early August for cv. Savatiano, and the second spraying was applied 20–30 days before harvest for both varieties. For the IPM schemes, the first spraying was carried out with the chemical PPPs when the grapes of each variety were at veraison, and the second spraying was carried out with the combination of biological PPPs, 20–30 days before harvest for both varieties. Mock vines were sprayed with water. Grapes in experimental plots were naturally infected by *A. carbonarius.* The biological PPP Mycostop^®^ was evaluated only for the year 2016 due to its commercial unavailability in the following years. Some PPPs (Scala^®^, Remedier^®^, Mevalone^®^ and Tusal^®^) were introduced in later years and were evaluated for two or three years.

#### 5.1.2. Disease Assessment

Grapes from the experimental plots were harvested. Disease severity was assessed by estimating the average percentage of diseased berries per bunch, calculated across all bunches collected for each repetition of the treatment application.

#### 5.1.3. Ochratoxin A (OTA) Quantification

Post-harvest, grape berries for each treatment were homogenized and OTA was extracted using 70% methanol. Analysis of the produced OTA was then carried out by the ELISA method using an Elisa AgraQuant^®^ ochratoxin A assay kit (2–40 µg kg^−1^) (Romer Labs, Getzersdorf, Austria) (LOD = 1.1 µg kg^−1^ for grape must). Absorbance was measured using a BioTek ELx800 reader obtained from Agilent distributor.

### 5.2. Sensitivity Tests on Active Substances

To assess the sensitivity of Greek *A. carbonarius* strains, seven active ingredients were selected that were registered for vines. Azoxystrobin, boscalid, chlorothalonil, cyprodinil, fludioxonil, pyrimethanil and tebuconazole are components of fungicides belonging to various chemical groups commonly used in viticulture and were also used in the experiments on the vineyards located in Spata. The chosen active substances employed were of analytical grade purity and were applied in concentrations that are presented in Table 4. The *A. carbonarius* strains used in the sensitivity tests (nine strains) were chosen based on their high ochratoxigenic ability. The origins and years of isolation of the isolates are presented in Table 5.

#### 5.2.1. Mycelial Growth Inhibition

The selected strains were grown on Malt Extract Agar (MEA, LabM) at 28 °C. After five days, 3 × 3 mm sections were excised from the periphery of the growing mycelium for each strain and transferred to Water Agar (WA). On the second day of incubation on WA, PDA plates (Potato Dextrose Agar) containing the different concentrations of each active substance were inoculated with 3 × 3 mm WA sections containing fungal hyphae. Each plate received 20 mL of PDA. Three replicates were performed for each strain and active substance. The plates were incubated at 28 °C for 5 days. Colony diameter (cm) measurements were taken from the third to the fifth day of incubation. Control plates, containing only the nutrient substrate, were included. Measurements from the fifth day were used to calculate the percentage of diameter inhibition, and results were expressed accordingly. Subsequently, the susceptibility of the nine *A. carbonarius* isolates was determined using EC_50_ values, calculated using Statgraphics^®^ software (STATGRAPHICS Centurion XVI, Version 16.1.11).

#### 5.2.2. Conidial Germination Inhibition

The strains were cultivated on PDA. After five days, conidia were collected from each strain and a conidial suspension was created with a final concentration of 10⁵ conidia mL^−1^. Subsequently, PDA plates containing 10 mL of PDA and the respective concentrations of each active substance received 100 μL of the conidial suspension for each strain that was spread on the surface of the media. The plates were incubated at 28 °C for 24 h, and the percentage of germinated conidia was determined. Control plates, containing only the nutrient substrate, were included. Three replicates were conducted for each concentration. The relative inhibition of germinated conidia compared to the control was calculated. EC_50_ values were determined for each concentration of the active substances used. EC_50_ values were calculated using Statgraphics^®^ software.

### 5.3. Statistical Analyses

All the data were normalized by square-root transformation and then subjected to ANOVA followed by Tukey’s Honestly Significant Difference (HSD) test as a post hoc test (StatGraphics Software). Differences at *p* ≤ 0.05 were considered significant.

## Figures and Tables

**Table 1:** EC_50_ values for each of the nine *A. carbonarius* strains and each compound (μg mL^−1^).

Strains	Boscalid	Azoxystrobin	Fludioxonil	Cyprodinil	Pyrimethanil	Tebuconazole	Chlorothalonil
AASN54	0.1934	0.1111	<0.1	0.0128	0.0433	0.8751	0.1937
BASN31	0.1237	<0.025	<0.1	0.0111	0.0430	0.2999	1.0189
CASN16	0.2073	0.0394	<0.1	<0.01	0.0457	0.3260	0.1398
DASN43	0.1487	0.0453	<0.1	0.0107	0.0845	0.4344	0.6098
EASN14	>5	0.0740	<0.1	<0.01	0.1862	0.2619	0.6339
AC33	3.3275	0.0936	<0.1	0.0102	0.0414	0.2718	0.0833
AC29	<0.1	<0.025	<0.1	<0.01	0.0426	0.1308	0.1608
AC55	2.7692	<0.025	<0.1	<0.01	0.0398	0.0862	0.0283
AC60	0.1580	0.0298	<0.1	<0.01	0.0475	0.3858	0.0412

**Table 2:** List of chemical plant protection products (PPPs).

Chemical PPPs	Active Substances	Maximum Certified Dose	Supplier
Cantus^®^	Boscalid	12 gr L^−1^	BASF Hellas S.A., Athens, Greece
Chorus^®^	Cyprodinil	5 gr L^−1^	Syngenta Hellas S.A., Athens, Greece
Geoxe^®^	Fludioxonil	1 gr L^−1^	Syngenta Hellas S.A., Athens, Greece
Quadris^®^	Azoxystrobin	1 mL L^−1^	Syngenta Hellas S.A., Athens, Greece
Scala^®^	Pyrimethanil	2 mL L^−1^	Bayer Hellas A.G., Athens, Greece
Switch^®^	Fludioxonil + cyprodinil	1 gr L^−1^	Syngenta Hellas S.A., Athens, Greece

**Table 3:** List of biological plant protection products (PPPs) and biocontrol agents.

Biological PPPs/ Biocontrol Agents	Active Substances	Maximum Certified Dose	Supplier
Botector^®^	*Aureobasidium pullulans* DSM 14940/DSM 14941	1 gr L^−1^	Elanco Hellas S.A., Athens, Greece
EM^®^	Effective microorganisms: mixture of beneficial microorganisms	0.2 mL L^−1^	EMRO, Japan, Okinawa, Japan
Mevalone^®^	Eugenol, geraniol and thymol	4 mL L^−1^	K & N Efthymiadis S.A., Athens, Greece
Mycostop^®^	*Streptomyces griseoviridis* strain K61	1 gr L^−1^	Verdera Oy Finland, Espoo, Finland
Remedier^®^	*Trichoderma asperellum* ICC012 *Trichoderma gamsii* ICC080	2.5 gr L^−1^	Agrology S.A., Thessaloniki, Greece
Serenade Max^®^	*Bacillus amyloliquefaciens* strain QST 713	4 gr L^−1^	Bayer Hellas A.G., Athens, Greece
Trianum^®^	*Trichoderma harzianum* T22	0.3 g L^−1^	Koppert B.V. Hellas, Athens, Greece
Tusal^®^	*Trichoderma asperellum* T25 *Trichoderma atroviride* T11	1 gr L^−1^	K & N Efthymiadis S.A., Athens, Greece
Vacciplant^®^	Laminarin	2 mL L^−1^	Alfa Agricultural Supplies S.A., Athens, Greece
Y1	*Aureobasidium pullulans*	10^7^ cfu mL^−1^	Plant Pathology—AUA, Athens, Greece
Mix Yeasts	*Candida railenensis* Z8 *Aureobasidium pullulans* Z31 *Aureobasidium pullulans* Y1 *Rhodotorula mucilaginosa* SR8 *Debaryomyces hansenii* VOL3	10^7^ cfu mL^−1^	Plant Pathology—AUA, Athens, Greece

**Table 4:** The active substances selected with their corresponding concentrations.

Active Substances	Applied Doses (μg mL^−1^)					
Azoxystrobin	0.025	0.05	0.1	0.5	1	5
Boscalid	0.1	0.5	1	5		
Chlorothalonil	0.01	0.1	0.5	1	5	
Cyprodinil	0.01	0.1	1	10	50	
Fludioxonil	0.1	1	10	50		
Pyrimethanil	0.01	0.025	0.05	0.1	0.5	1
Tebuconazole	0.05	0.1	0.5	1	5	

**Table 5:** *Aspergillus carbonarius* strains used in sensitivity tests, years of isolation and areas of origin.

Year of Isolation	Strain Code	Origin	Reference *
2015	AASN54	Stimagka	PP AUA
2016	BASN31	Stimagka	PP AUA
2017	CASN16	Spata	PP AUA
2018	DASN43	Spata	PP AUA
2019	EASN14	Spata	PP AUA
2014	AC29	Crete	[57]
2014	AC33	Attica	[57]
2014	AC55	Peloponnese	[57]
2014	AC60	Macedonia	[57]

* PP AUA: Laboratory of Plant Pathology, Agricultural University of Athens.

## Data Availability

The datasets generated during and/or analyzed during the current study are available from the corresponding author on reasonable request. The data are not publicly available due to potential further exploitation actions.
